# Distribution of Hepatitis C Virus Genotypes Among Azerbaijani Patients in Capital City of Iran-Tehran

**DOI:** 10.5812/hepatmon.13699

**Published:** 2013-09-01

**Authors:** Farah Bokharaei-Salim, Hossein Keyvani, Seyed Hamidreza Monavari, Seyed Moayed Alavian, Shahin Fakhim, Sherko Nasseri

**Affiliations:** 1 Department of Virology, Iran University of Medical Sciences, Tehran, IR Iran; 2 Baqiyatallah Research Center for Gastroenterology and Liver Diseases, Baqiyatollah University of Medical Sciences, Tehran, IR Iran; 3 Middle East Liver Disease Center, Tehran, IR Iran; 4 Faculty of Engineering Deaprtment, Islamic Azad University, Shahre Qods Branch, Tehran, IR Iran; 5 Department of Virology, Tehran University of Medical Sciences, Tehran, IR Iran

**Keywords:** Hepatitis C, Infection, Genotype

## Abstract

**Background:**

Determination of the Hepatitis C virus (HCV) genotype distributed in a particular area has an important role on public health throughout the world.

**Objectives:**

The aim of this study was to determine the frequency of HCV genotypes in Azerbaijani patients.

**Patients and Methods:**

From March 2010 until March 2012, 235 Azerbaijani patients with established chronic hepatitis C, referred to Hospitals related to Iran University of Medical Sciences and Tehran Hepatitis Center, Clinical department of Baqiyatallah Research Center for Gastroeneterology and Liver Disease, were enrolled in this cross sectional study. About 5 mL of peripheral blood was collected from patients and after separation of plasma, viral RNA extracted. HCV-RNA were amplified by RT-nested PCR using primers from the 5´-UTR and genotyped by RFLP assay, and then HCV genotypes were confirmed using sequencing of cloned PCR products into pJET1.2/blunt cloning vector.

**Results:**

HCV genotyping of positive plasma samples demonstrated that predominant HCV subtype was noted for 1b (71.1%) followed by subtype 3a (17.0%), genotype 2 (6.8%), 1a (1.7%), and mixed infection (3.4%). The mean ± SD age of patients was 37.3 ± 11.8 (range: 2-63) years. Out of 235 patients, 139 (59.1%) were male. The frequency of HCV subtype 3a was higher in patients under 40 years old (3a: 18.1% vs. 15.0%), and subtype 3a was higher in male patients (3a: 18.7% vs. 14.6%).

**Conclusions:**

The current study shows that the predominant HCV genotype among Azerbaijani patients with established chronic hepatitis C is subtype 1b (71.1%) followed by subtype 3a (17.0%).

## 1. Background

Hepatitis C Virus (HCV) is an enveloped positive sense, single stranded RNA virus which belongs to the family Flaviviridae and genus hepacivirus (1). According to Simmonds nomenclature, HCV strains are classified into six distinct virus genotypes (1 to 6) and more than 70 subtypes (e.g., subtype 1a, 1b) (2). Hepatitis C is a major health problem affecting approximately 3% of the world population (about 170 million people) (3) and it is an agent for acute, chronic and fulminate hepatitis (4). Nearly 25% of patients with hepatitis C virus infection develop cirrhosis and hepatocellular carcinoma (3).

Hepatitis C virus genotyping is an important tool in management of the HCV infected patients and in the epidemiology (5). Importantly, great numbers of studies have shown a relationship between the HCV genotype and the response to interferon (IFN) and pegylated interferon (Peg-IFN) therapy in combination with ribavirin (6). Therefore the HCV genotypes should be determined prior to antiviral therapy, because it can provide clinically valuable information that can be used to direct the duration and type of antiviral therapy and also to predict the possibility of sustained HCV clearance after antiviral therapy (6, 7).

It should also be noted that the HCV genotypes are distributed differently and have variant susceptibility to antiviral therapies (3). Some studies have revealed that HCV infected patients with genotype 2 and genotype 3 have a sustained a better response to antiviral therapy (65%) than HCV infected patients with genotype 1 (30%) (8).

The distribution of HCV genotypes vary from country to country, for instance HCV genotypes 1, 2 and 3 are distributed widely around the world, while the most common genotypes in the United States and Europe are HCV subtypes 1a and 1b (9, 10).

The most prevalent HCV genotype in North Africa and the Middle East is genotype 4 (7) and in South Africa and Southeast Asia are genotypes 5 and 6 ,respectively (3, 11).

The prevalence of HCV genotypes were found to be 1a (47%) and 3a (36%) in Iran (12). The predominant HCV genotype is 1b in Turkey, Russia, Belarus, Moldova, Uzbekistan, Lithuania, and Latvia (13-17), and genotype 3a in Pakistan (7, 18).

## 2. Objectives

In Republic of Azerbaijan, no study has been done in order to determine the geographical distribution of different HCV genotypes; therefore the prevalence of various HCV genotypes is unknown in this region. The purpose of this study was to determine the prevalence of HCV genotypes in Azerbijani patients with established chronic hepatitis C.

## 3. Material and Methods

### 3.1. Study Population

The current cross sectional study was conducted on 235 consecutive Azerbaijani patients with established chronic hepatitis C (the patients come from Republic of Azerbaijan country to Iran for medical treatment) who had been selected for anti-hepatitis C treatment referred to Hospitals related to Iran University of Medical Sciences and Tehran Hepatitis Center, Clinical department of Baqiyatallah Research Center for Gastroeneterology and Liver Disease, from March 2010 to March 2012. Precipitants were informed about the study and a written consent form was obtained from each patient. The current study was approved by the local ethics committee of Gastrointestinal and Liver Disease Research Center (GIDRC) of Iran University of Medical Sciences.

### 3.2. Collection and RNA Extraction of Samples

Five milliliters of peripheral blood was taken from each patient into EDTA-containing vacationer tubes. Plasma was separated from whole blood and immediately stored at -80°C. Viral RNA was extracted from 140 µL of plasma using a commercial kit (Qiagen GmbH, Hilden, Germany) according to the manufacturer’s recommendations.

### 3.3. cDNA Synthesis and HCV Genotyping

For cDNA synthesis, 10 µL of extracted RNA was added to reaction mixture which contained 4 μl of 5X reverse transcriptase reaction buffer, 200 U of Moloney Murine Leukemia Virus Reverse Transcriptase (Fermentas GmbH, St. Leon-Rot, Germany), 125 µmol mix deoxynucleotidetriphosphat, 8 units of RNase inhibitor (Fermentas GmbH, St. Leon-Rot, Germany), 20 pmol of random hexamer, as well as 1 μL of diethyl-pyrocarbonate (DEPC) treated water. The reactant was incubated at 42 °C for 30 min and then at 72 °C for 10 min, when the reverse transcriptase, inactivated. The guidelines of Kwok and Higuchi (19) were completely followed to avoid carryover any contamination and appropriate positive and negative controls were routinely used in all steps (RNA extraction, cDNA synthesis and each round of PCR).

Both nested-polymerase chain reaction (Nested-PCR) and restriction fragment length polymorphism (RFLP) assay were performed using primers from the 5´-untranslated region (5´-UTR) as described by Pohjanpelto et al (20).

Undigested PCR products (173-bp) of specimens with digested PCR products by restriction enzymes, positive and negative controls and 50bp molecular weight marker (Fermentas GmbH, St. Leon-Rot, Germany) were visualized by 3% agarose gel electrophoresis. HCV Genotypes were determined based on fragmentation pattern of the amplified DNA.

To confirm the results of HCV-genotyping by RFLP assay, the 5´-UTR region of HCV from 6 randomly selected specimens were amplified with Pfu DNA polymerase and then PCR products from the second round of nested-PCR were cloned into pJET1.2/blunt cloning vector (Fermentas GmbH). The DNA from two clones of each specimen was sequenced by dye termination method using the ABI 3730 XL sequencer.

### 3.4. Statistical Analysis

Statistical analysis was performed by SPSS version 17 (SPSS, Chicago, IL). Descriptive analyses as well as Student’s t-test, Mann Whitney and Chi-square as well as Fisher’s exact test were used. A (P < 0.05) was considered statistically significant.

## 4. Results

A total of 235 patients with established chronic hepatitis C were enrolled in this cross sectional study. The HCV genotype of the study population was carried out before starting antiviral treatment. The HCV genotypes frequency was determined as follows: genotype 1b in 165 (71.1%) patients, genotype 3a in 40 (17.0%), genotype 2 in 16 (6.8%) patients, 1a in 4 (1.7%), and mixed infection in 8 (3.4%) patients. Of 8 mixed genotype infection patients, mixed inter-genotype infection 1b and 3a was the most common (62.5 %). It should also be noted that mixed HCV infection was detected in 6 (75%) of patients with multi blood or blood products transfusion that was statistically significant (P value = 0.031). The HCV genotypes of the patient's plasma sample were confirmed via nucleotide sequencing of the HCV 5′-UTR. Demographic data and distribution of HCV genotype in all the patients are presented in Table 1. 

The mean ± SD age of patients was 37.3 ± 11.8 (range: 2-63) years. Out of 235 patients, 139 (59.1%) were male. The mean age of the women was statistically higher than men (39.6 vs. 35.7, respectively) (T-test, P < 0.05). The frequency of the genotype 1b in participants under and above 40 years old was 71.6% and 70.0% respectively, which was not statistically significant. The frequency distribution of HCV subtype 3a was higher in patients younger than 40 years old (3a: 18.1% vs. 15.0%) (T-test, P < 0.05), and subtype 3a distribution was higher in male patients (3a: 18.7% vs. 14.6%) that was statistically significant (T-test, P < 0.05).

**Table 1.** Demographic Parameters and Hepatitis C Virus Genotypes Distribution among Azerbaijani Patients in Capital City of Iran-Tehran

Parameters	Female	Male	Total
**No. (%)**	96 (40.9)	139 (59)	235 (100)
**Mean age**	39.6 ± 13.3	35.7 ± 10.1	37.3 ± 11.8
**Type of HCV Genotype and Subtypes **			
1a	2 (2.1)	2 (1.7)	4 (1.7)
1b	70 (72.9)	97 (69.8)	165 (71.1)
2	7 (7.2)	9 (6.5)	16 (6.8)
3a	14 (14.6)	26 (18.7)	40 (17.0)
Mixed infection	3 (3.1)	5 (3.6)	8 (3.4)
**HCV Mixed Infection**			
1a and 3a	−	1 (0.7)	1 (0.4)
1b and 2	−	1 (0.7)	1 (0.4)
1b and 3a	3 (3.1)	2 (1.4)	5 (2.1)
2 and 3a	−	1 (0.7)	1 (0.4)

## 5. Discussion

The worth of HCV genotyping as an epidemiological parameter has been shown. The present study was performed on 235 chronically HCV infected Azerbaijani patients, who come from Republic of Azerbaijan country to Iran for medical treatment, to determine the prevalence of HCV genotypes in their plasma specimens. The frequency of HCV genotypes was found as follows: HCV genotype 1b was the most frequent (71.1%), followed by genotype 3a (17.0%), genotype 2 (6.8%), genotype 1a (1.7%), and multiple HCV genotypes in 3.4% of the patients.

Little is known about the distribution of HCV genotypes in the former Soviet Union, where hepatitis C is endemic (17). This is the first study conducted in Azerbaijani patients; therefore we are not able to compare the results with that. There are some reports about the prevalence of HCV genotypes in the former Soviet Union, which are compatible with the current study. The most prevalent HCV genotype in different regions of the former Soviet Union is genotype 1b: Russia (76%), Moldova (89%) (17), Belarus (53.8%) (21), Uzbekistan (64.2%) (22), Estonia (71%) (23), Lativa (85%) (24), Lithuania (54%) (25), Georgia (59%) (26), and Tajikistan (84%) (27), (Table 2), that are comparable with the present study’s result. The distribution of HCV genotypes and subtypes in non-Arab countries (14 , 28 , 29), and Arab countries (25, 30-33) in the Middle East and several countries of the former Soviet Union (17, 21-25) are shown in Table 2. 

**Table 2.** Distribution of Hepatitis C Virus Genotypes and Subtypes in the Former Soviet Union and the Middle East

Region/ Countries	Genotype and Subtype		References
	Most Common	Less Common	
**The former Soviet Union**			
Russia	1b (76.0)	3a (13.0), 2a (7.0)	(17)
Estonia	1b (71.0)	3a (24.0)	(34)
Uzbekistan	1b (64.2)	3a (25.0)	(22)
Belarus	1b (53.8)	3a (38.5), 1a (5.1)	(21)
Moldova	1b (89.0)	2 (4.0)	(17)
Latvia	1b (85.0)	3a (10.0)	(35)
Lithuania	1b (54.0)	3a (23.9), 2a (10.9), 2b (4.4)	(36)
Georgia	1b (59)	3a 27), 2a/2c (11), 1a (3)	(26)
Tajikistan	1b (84.6)	3a (7.6), 2a (5.7, 2c (1.9)	(27)
**Non-Arab Countries**			
Iran	1a (39.7)	3a (27.5), 1b (12.1)	(29)
Turkey	1b (87.2)	1a (9.9)	(14)
Pakistan	3a (54.4)	3b (8.2), 1a (6.8), 1b (4.6), 4 (1.3)	(28)
**Arab Countries**			
Saudi Arabia	4 (62.0)	1 (24.0), 2 (7.4)	(33)
Jordan	1a (40.0)	1b (33.3), 4 (26.7)	(25)
Kuwait	4(38.0)	1(27.0)	(32)
Iraq	4	1b, 1a, 3a	(37)
UAE	4 (45.4)	3a (23.8), 1a (15.0)	(30)
Oman	4	Not available	(37)

It was estimated that the divergence of different variants of subtype 1b has occurred about 70-80 years ago (39). Regarding the isolation of the former Soviet Union from the other parts of the world after the revolution of Bolsheviksin 1917, it is be possible that the time of divergence of HCV variants of subtype 1b, in that region goes beyond 80 years. Because of isolation of the former Soviet Union, the predominant HCV genotype is subtype 1b in different countries in this region (17). Noteworthy is the fact that the subtype 1b was spread principally through blood transfusion or blood products and medical procedures (17, 22), whereas there were reports from St. Petersburg, Russia, and from several European countries that found much more often a high prevalence of subtype 3a among intravenous drug users than in general population (38, 40). Interestingly there are some reports that show a different distribution of HCV genotypes during years, for instance; prodominant HCV genotypes was 1b (90.0%), and 3a (10.0%) in 1997 (17), and 1b (64.2%), and 3a (25%) in 2003 in Uzbekistan (22), and was 1b (70%), and 3a (20.0%) in 1997 (17), and 1b (53.8%), 3a (38.5%), and 1a (5.1%) in 2008 in Belarus (21). These reports might show a changing in the distribution of HCV genotypes in these countries that needs more investigation. On the other hand the most frequent of HCV genotypes was genotype 1b (71.1%), 3a (17.0%), and 2 (6.8%) in the present study. Due to lack of information, we could not compare the results. Therefore, it seems that more studies focusing on determination of the prevalence of HCV genotypes in different population will be needed in Azerbaijani HCV infected patients.

In the present study we found that the frequency of HCV subtype 3a was higher in patients younger than 40 years old (3a: 18.1% vs. 15.0%) that was statistically significant. This finding is compatible with some recent studies that show an increase in the distribution of HCV subtype 3a in the young population of Iran (41), Germany (42), and Slovenia (43).

In conclusion, the current study indicates that the predominant HCV genotype among Azerbaijani patients with established chronic hepatitis C is subtype 1b (71.1%) followed by subtype 3a (17.0%), and 2 (6.8%). This is a preliminary study and a study with large population size is required to determine of HCV genotype in different population in Republic of Azerbaijan country.
